# Structure-based modelling of hemocyanin allergenicity in squid and its response to high hydrostatic pressure

**DOI:** 10.1038/srep40021

**Published:** 2017-01-23

**Authors:** Yifeng Zhang, Yun Deng, Yanyun Zhao

**Affiliations:** 1 Key Laboratory of Urban Agriculture (South), Ministry of Agriculture, SJTU-Bor S. Luh Food Safety Center, Department of Food Science and Technology, Shanghai Jiao Tong University, 800 Dongchuan Road, Shanghai 200240, China; 2 Department of Food Science & Technology, Oregon State University, 100 Wiegand Hall, Corvallis, OR 97331, USA

## Abstract

The secondary, tertiary, and quaternary structures of squid hemocyanin (Hc) were characterised, and the relationship between Hc structure and allergenicity responses to high hydrostatic pressure (HHP) was modelled. The Hc allergenicity varied with its protein structure. Electrophoresis analysis revealed that HHP treatment significantly decreased the band intensity of Hc when increasing pressure from 200 and 400 MPa to 600 MPa. The protein structure analysis of squid Hc showed that while HHP treatment decreased the α-helix content, free sulfhydryl content, and R_g_, it increased the random coil content, surface hydrophobicity index (Ho), Guinier aggregation number (〈N_agg_〉_G_) and average aggregation number (〈N_agg_〉_Q_). The α-helix and random coil contents of the 600 MPa treated samples were 23.67% and 37.54%, respectively, compared to 32.37% and 32.02% in the control, respectively. HHP treatment decreased the IgE and IgG-binding capacities, indicating a significant decrease in the allergenicity (*P*< 0.05) of squid Hc. This study provided meaningful information of applying HHP to reduce allergenicity, and explained the responses of Hc protein structure to HHP for lowering the allergenicity of squid.

Seafood allergy is one of the most frequent and severe food allergies, and it induces some symptoms, such as angioedema, gastrointestinal distress, asthma, rhinitis, and anaphylaxis in individuals^1^,^2^. Hemocyanin (Hc) has been identified as one of the major allergens of squid (*Todarodes pacificus*) and is also the major allergen in diverse seafood^1^,^2^. Hc is one of the large, multi-subunit copper proteins that function as oxygen transporter by dissolving freely in the haemolymph in many species^3^. Molluscan Hc forms a decamer (cephalopods) or multi-decamer (gastropods) and contains 7–8 functional units^4^. Hc has a large and complex structure^5^, which has been characterized by several methods, such as transmission electron microscopy (TEM) and X-ray diffraction (XRD)^5^. However, the protein structure (secondary, tertiary, and quaternary structure) of squid Hc is still unclear. Furthermore, the relationship between the allergenicity and the protein structure of Hc has rarely been reported.

The allergenicity of proteins is affected by the changes in the secondary and tertiary structure of protein^6^,^7^. For example, the allergenicity of soy protein decreased by changing the secondary structure and partial unfolding simultaneously^8^. The decreased allergenicity and IgE-binding activity of bovine gamma globulin were attributed to the changes in the tertiary structure caused by pressurization^9^. Moreover, an increase in the allergenicity of β-lactoglobulin was caused by the unfolding of the protein and the exposure of hidden epitopes^10^. In addition, the quaternary structure of proteins probably played an important role in allergenicity for the location of the IgE-binding epitopes and the presence of digestion resistant fragments, such as protecting these epitopes from digestive enzymes^11^,^12^. It has been reported that quaternary structure of Ara h 1, forming trimers, enhanced its allergenicity^11^. Nevertheless, no clear relationship between the quaternary structural parameters and allergenicity has been established.

Small-angle X-ray scattering (SAXS) has been used to determine the structure of biological macromolecules in solutions^13^. SAXS has also been often used to study the quaternary structure of a protein^13^,^14^. A significant progress has been made over the years in using SAXS to obtain the quaternary structural information of Hc obtained from different types of species^14^,^15^,^16^. The SAXS-based evidence indicated that the conformation of oxy-Hc (*Octopus vulgaris*) differs from that of the deoxy-form in the case of both molluscan and arthropod Hc. The SAXS-based three-dimensional reconstruction of the immunogen KLH1 showed different oxygen dependent conformations^14^.

Our previous studies proved that high hydrostatic pressure (HHP), a valuable non-thermal food processing technology, can significantly decrease the allergenicity of squid tropomyosin by modifying the secondary and tertiary structures of protein^6^,^7^. Hence, one objective of this study was to evaluate the effect of different HHP treatments on the allergenicity of squid Hc samples since this is important for the comprehensive assessment of seafood allergy. HHP treatments affect the structure of proteins, thus reducing their allergenicity^6^,^7^. However, limited information is available to establish the relationship between allergenic properties and protein structure. Mathematical modelling could be used to visualize the complicated biochemistry changes and relationships. For example, mathematical model was used to analyse the treatment efficacy for prostate cancer^17^ and to evaluate the efficacy of vaccines for control of liver fluke infection^18^. To the best of our knowledge, limited information has been reported about the complex structure-allergenicity changing process in the allergenic protein. Hence, the mathematical modelling may be used for understanding this intricate change.

The other objectives of this study were to investigate the structural properties (secondary, tertiary, and quaternary structure by SAXS) and allergenicity (indirect ELISA, using the human sera allergic patients or rabbit anti-squid Hc polyclonal antibodies) of HHP-treated squid Hc. Finally, a mathematical model between the allergenicity and protein structure parameters in HHP-treated squid Hc samples was developed.

## Results

### Identification of squid Hc

**Figure 1:**
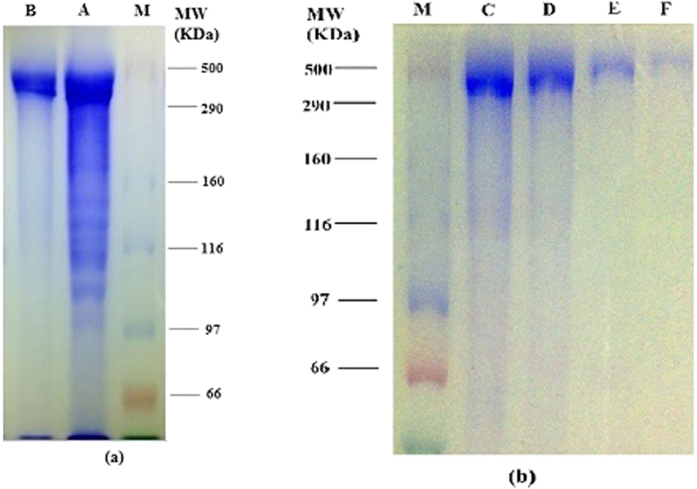
Electrophoresis (SDS–PAGE) analysis of different squid hemocyanin samples. (**a**) Hc purification (M: marker; A: crude Hc, after treating with 67% ammonium sulfate solutions; B: purified Hc, after isoelectric precipitation). (**b**) Untreated and different HHP-treated Hc samples (M: protein markers; C: untreated Hc; D, E, and F samples were processed at 200 MPa, 400 MPa, and 600 MPa for 20 min, respectively).

As shown in Fig. 1a-A, the squid Hc samples were analysed by sodium dodecyl sulfate polyacrylamide gel electrophoresis (SDS-PAGE); a band with an average MW of 385 kDa was clearly observed in the crude Hc. After the purification by ammonium sulphate precipitation (67% saturation) and centrifugation at 11,300 r/min for 60 min, the purified Hc on SDS-PAGE showed a single band with an average MW of 385 kDa (Fig. 1a-B), indicating that the protein was pure. The squid Hc sample was identified using the MASCOT search engine, and the LC-Q-TOF-MS fit is reported in Table 1. These results clearly indicated that the purified protein was Hc, and it could be used for further studies.

### SDS-PAGE

Figure 1b shows the SDS-PAGE pictures of the control and different HHP-treated squid Hc samples. SDS-PAGE is usually performed to determine the presence or absence of protein allergens^7^. The band of Hc was observed at 385 kDa in all the HHP-treated samples. However, the band intensities of Hc decreased along with the increase in the HHP from 200 MPa to 600 MPa. This was related to the changes in the molecular structure, indicating different responses to different pressure levels^19^.

### Secondary structure

**Figure 2:**
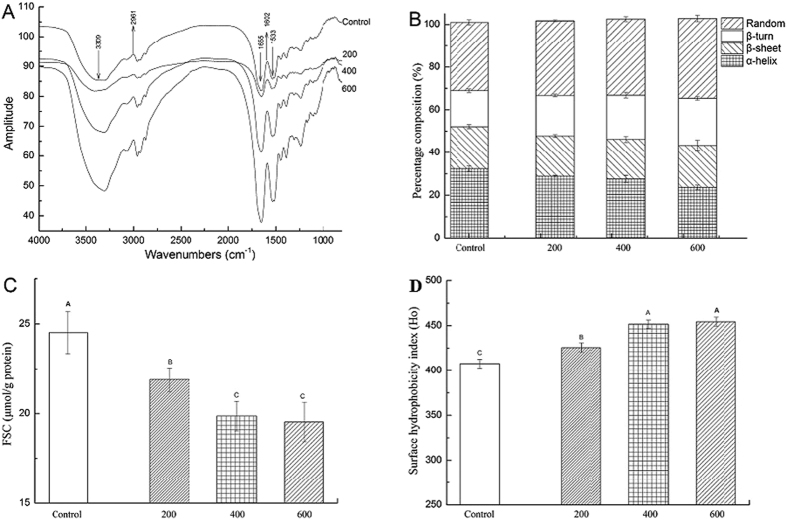
FTIR spectrum (**A**), secondary structure (**B**), and tertiary structure (**C,D**) of different HHP-treated squid Hc samples. (**B**) α-helix, β-sheet, β-turn, random: percentage composition (%) of secondary structure from FTIR spectrum. (**C**) FSC, μmol/g protein and (**D**) Ho indexes were 200, 400, and 600 for the HHP treatments maintained at 200 MPa, 400 MPa, and 600 MPa for 20 min, respectively.

The structural properties of protein are directly related to its molecular structure and allergenic properties^20^. Figure 2A reports the results of Fourier transform infrared (FTIR) spectroscopy analysis, providing information about the secondary structural characteristics of squid Hc. As shown in Fig. 2A, the HHP-treated samples and control had similar FTIR spectra. All the squid Hc samples clearly showed positive peaks at 2961 cm^−1^ and 1602 cm^−1^ and negative peaks at 3309 cm^−1^, 1665 cm^−1^, and 1533 cm^−1^. The amide I (1600–1700 cm^−1^) band is widely used in the analysis of protein secondary structure including α-helix (peaks at 1654 ± 4 cm^−1^), β-sheet (peaks at 1620 ± 20 cm^−1^), β-turn (peaks at 1680 ± 20 cm^−1^), and random coils (peaks at 1645 ± 5 cm^−1^) in the fitting procedure^21^,^22^. Those peaks in Amide-I band (1600–1700 cm^−1^) and Amide-II band (1500–1600 cm^−1^) are very important. In this study, the peaks at 1602 cm^−1^ was probably attributed by the coordinated NH_3_^21^, and the band of approximately 1665 cm^−1^ in FTIR spectroscopy was corresponding to the β-turn of secondary structure^21^,^22^,^23^.

The effects of HHP treatments on the secondary structure of squid Hc are illustrated in Fig. 2B. Clearly, the HHP treatments significantly changed the secondary structure of squid Hc. The initial secondary structure of the control squid Hc sample was composed of 32.37% α-helix, 19.77% β-sheet, 16.81% β-turn, and 32.02% random coils. With increasing pressure, the content of α-helix decreased from 32.37% in the control to 23.67% in the 600 MPa treated protein, while the contents of β-turn and random coils increased from 16.80% to 21.95%, 32.02% to 37.54%, respectively. However, there was no significant difference in β-sheet content among the samples. These results suggested that HHP induced a conversion of α-helix to β-turn and random coils. The α-helix content in squid tropomyosin was significant embodiment for the allergenicity of squid tropomyosin^6^,^7^.

### Tertiary structure

Free sulfhydryl content (FSC) and surface hydrophobicity index (Ho) were used to evaluate the tertiary structure of squid Hc. The disulfide bonds play an important role in defining the tertiary structure of protein^24^. Ho represents the number of hydrophobic groups on the surface of a protein and changes with protein conformation^25^. Ho has been used to evaluate the conformational difference in the tertiary structure of a protein^26^ and is closely related to its allergenicity property^27^. The FSC and Ho of the tertiary structure in different HHP-treated squid Hc samples are exhibited in Fig. 2C and D, respectively. Generally, the HHP treatments significantly (*P* < 0.05) decreased the FSC with increasing pressure, from 24.52 μmol/g protein (control) to 19.52 μmol/g protein (600 MPa). As shown in Fig. 2D, clearly the higher the pressure, the higher the Ho for HHP-treated squid Hc. However, no significant difference was observed between 400 MPa and 600 MPa treated samples for both FSC and Ho in this study.

### Quaternary structure determination by SAXS

**Figure 3:**
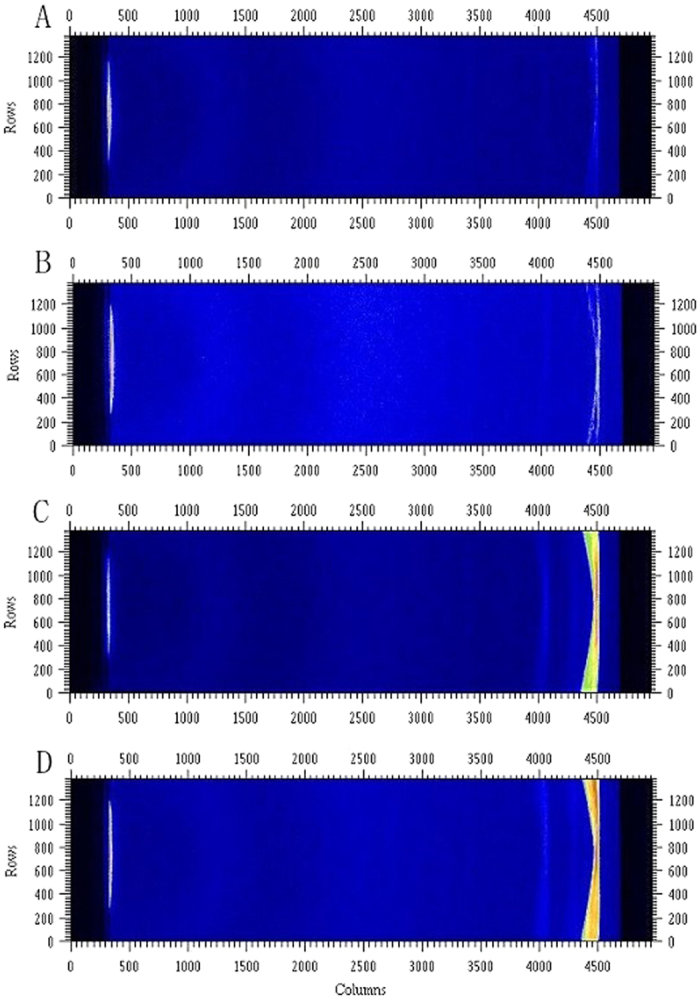
SAXS images of squid Hc under different HHP treatment conditions. (**A**) untreated squid Hc; (**B–D**) HHP-treated at 200 MPa, 400 MPa, or 600 MPa for 20 min, respectively.

The SAXS images illustrated the structural differences among the squid Hc samples subjected to different HHP treatments (Fig. 3). To better understand the SAXS results, the SAXS parameters R_g_,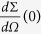, 〈N_agg_〉_G_, and 〈N_agg_〉_Q_ of different HHP-treated squid Hc samples were considered. These four parameters are the most commonly used descriptors of SAXS, in which R_g_ is the particle gyration radius; 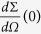 is the forward scattering cross-sections; 〈N_agg_〉_G_ is the Guinier aggregation number; and 〈N_agg_〉_Q_ is the average aggregation number^13^,^14^,^15^. The SAXS images were analysed using the SAXS Quant 2.0^14^,^16^, and the SAXS parameters R_g_, 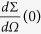, 〈N_agg_〉_G_, and 〈N_agg_〉_Q_ were calculated. The SAXS parameters of squid Hc under different HHP treatment conditions are represented in Table 2. In this study, the initial SAXS parameters R_g_, 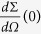, 〈N_agg_〉_G_, and 〈N_agg_〉_Q_ for the untreated (control) squid Hc were 142.66 ± 2.52, 28.67 ± 1.53, 10.20 ± 0.78, and 9.30 ± 1.14c, respectively, consistent with previously reported values^14^. Generally, the HHP treated squid Hc samples had lower R_g_ (134.33–112.34; *P* < 0.05) and 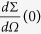 values (18.34–11.54; *P* < 0.05), but higher 〈N_agg_〉_G_ (11.91–16.03) and 〈N_agg_〉_Q_ values (12.67–15.23; *P* < 0.05) than control. Different values of SAXS parameters indicate different structural properties. The results shown in Table 2 also indicated that a higher pressure caused more changes in the SAXS parameters than a lower pressure at the same total pressure holding time (20 min).

### Allergenic properties

Indirect ELISAs were carried out to evaluate the allergenic properties of squid Hc using the human sera of five allergic patients (P_1_–P_5_) by determining the IgE-binding capacity (IgE) or rabbit anti-squid Hc polyclonal antibodies by determining the IgG-binding capacity (IgG). Table 3 reports the changes in the allergenic properties determined from the results of the indirect ELISAs performed with the squid Hc samples against IgE and IgG. In general, the HHP treatments decreased the IgE and IgG values compared to the control, indicating a decrease in the allergenicity of squid Hc. For IgE, regardless of the differences among the individual sera, i.e., different specificities to squid Hc, a significant decrease in the IgE-binding reactivity was observed after the HHP treatment at 200 MPa. For example, the IgE-binding reactivity of the 200 MPa treated samples decreased by 40% for P_3_, from 0.77 (control) to 0.47 (200 MPa). As such, the IgG (R, R_SGF_, and R_SIF_) values clearly indicated that the HHP treatments significantly decreased the binding capacities compared to the control (Table 3). However, no significant difference was observed in both IgE and IgG between the 400 MPa and 600 MPa-treated samples.

### Mathematical model

A mathematical model was used to evaluate the changes in the allergenicity properties of different HHP-treated squid Hc. IgG-binding capacity, used to estimate the allergenicity in many studies^6^,^20^,^28^,^29^, was selected as the allergenicity index for the mathematical model. All the protein structural parameters (α-helix, β-sheet, β-turn, random, FSC, Ho, R_g_, 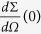, 〈N_agg_〉_G_, and 〈N_agg_〉_Q_) and pressure level in HHP treatments were also considered in the development of the mathematical model. The structure based allergenicity model can be described as following:


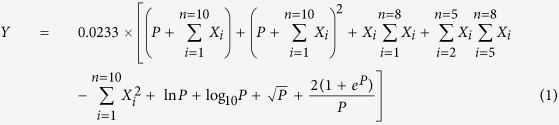


where Y was the indirect ELISA performed with rabbit anti-squid Hc polyclonal antibodies; P was the pressure: 0.1 MPa, 200 MPa, 400 MPa, or 600 MPa; X_1_–X_10_ were the values of α-helix, β-sheet, β-turn, random, FSC, Ho, R_g_, 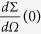, 〈N_agg_〉_G_, and 〈N_agg_〉_Q_, respectively. The correlation coefficient (R^2^), chi-square (χ^2^), mean relative deviation (MRD), and root mean square error (RMSE) were 0.98, 0.0051, 4.64%, and 6.30%, respectively.

## Discussions

The mathematical modelling approach was used to predict the reduced allergenicity of HHP-treated squid Hc with the change in protein structure. First, the SDS-PAGE analysis showed a single band with an average MW of 385 kDa (Fig. 1a-B), indicating that a purified protein was obtained. A similar band was observed by Matsuno *et al*.^5^; the Hc collected from the living squid (*T. pacificus*) was the only protein observed in the hemolymph (~100 mg/mL) by SDS–PAGE analysis. Hence, the purified protein could be used for further studies^5^.

The HHP treatments significantly decreased the band intensities of Hc with increase in the pressure level in HHP treatment from 200 MPa to 600 MPa in this study (Fig. 1b). Similar studies indicated that the 400 MPa and 600 MPa treated beef muscle samples exhibited significantly lower intensity (*P* < 0.001) in SDS-PAGE bands of ~26 KDa, 42 KDa, 48 KDa, 60 KDa and 63 KDa than control^30^,^31^. This decreased band intensities in SDS-PAGE could be related to the effect of HHP on the protein degradation^30^,^31^. Previous study demonstrated that HHP could be used to promote the proteolysis and enhance the proteolytic degradation^32^. The peptides of ovalbumin (such as YAEERYPIL, FRADHPFL and RADHPFL) were released under pressures of 200–400 MPa^32^. The 27 types of peptide segments (PS_1_-PS_27_, Supplementary Table S1) were analysed in this study (Supplementary information). The results also revealed that HHP could enhance the proteolytic degradation. The peptides of PS_22_-PS_27_ were cracked under pressures of 200–600 MPa. However, the degraded protein fragments or the released peptides caused by the HHP treatment were too weak for the resolution of the gel and ultimately lost in the buffer, and were invisible in the SDS-PAGE^17^,^33^,^34^. In this study, the cracked peptides in HHP treated Hc could be some of the reasons causing decrease in band intensities and allergenicity of Hc. For different HHP treatments, a higher pressure provided a higher energy, causing major changes in the molecular structure of squid Hc. Hence, the decrease in band intensities indicates changes in molecular structure and lesser availability for IgE binding. However, the exact mechanisms of lower intensity in SDS-PAGE bands and protein cleavage of squid hemocyanin caused by HHP are still not fully understood, thus requiring further studies.

For protein structure analysis, the values of α-helix content, FSC, R_g_, and 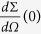 decreased, and the values of random coil content, Ho, 〈N_agg_〉_G_, and 〈N_agg_〉_Q_ increased in the HHP-treated squid Hc samples. Previous studies also reported that HHP treatments affect the secondary and tertiary protein structures^26^,^35^. The α-helix content in squid tropomyosin decreased with increasing pressure from 200 MPa to 600 MPa, and a modification of secondary structure decreased the allergenicity of squid tropomyosin^6^,^7^. The variations in the secondary structure of invertebrates’ tropomyosin were also related to the changes in allergenicity^7^,^26^,^35^. Moreover, sulfhydryl groups and disulfide bonds significantly affected the functional properties of food proteins and played an important role in the formation of relatively rigid structures, such as protein gels^36^. The results obtained in this study were consistent with those obtained previously^8^. For the decrease in FSC as the result of HHP treatment might be caused by the formation of disulfide bonds between the intramolecular and intermolecular protein chains through SH/S-S interchange reactions during HHP treatments^7^,^37^,^38^. The lower values of FSC with HHP treated samples were also reported in other allergens, such as squid tropomyosin^7^,^37^, soy protein isolate samples^38^ and β-lactoglobulin^39^, which was due to the formation of S-S bonds that was usually accompanied with hydrophobic interactions. These results indicate that HHP treatment unfolded the protein’s native structure, leading to the exposure of hydrophobic regions to the exterior of the protein molecules. However, this study shows that no significant difference was observed between the 400 MPa and 600 MPa treated samples for both FSC and Ho. The protein structure changed probably because of continuously applied pressure, powerful percussive action, and shear effect by compression and decompression^37^. The 400 MPa pressure might be sufficient to destabilize the intermolecular interactions and tertiary structure since no more change was observed when the pressure was increased to 600 MPa^8^,^27^.

The native structure of proteins changed after HHP treatments, and in turn these changes in secondary and tertiary structures may affect the SAXS parameters (quaternary structure). Previous atomic force microscopy (AFM) studies showed that HHP treatments cause changes in intermolecular structures (hydrogen bonds, ionic and hydrophobic interactions) and the surface topography, thus changing the protein structure^6^. When protein is subjected to HHP treatment, the water molecules squeeze into the free spaces between the amorphous lamellae and semi-crystalline regions because of strong forces produced by HHP^6^,^37^, indicating that the higher pressures cause more intermolecular changes (hydrogen bonds, ionic and hydrophobic interactions) in the squid Hc during the HHP treatments. However, the exact mechanisms of these changes in the protein structure in different HHP treatments are poorly understood, thus requiring more studies.

In this study, HHP treatments decreased the allergenicity of squid Hc. Previously, we reported that the allergenicity of squid tropomyosin significantly decreased after HHP treatments at 200 MPa, 400 MPa, or 600 MPa for 20 min^7^. The protein degradation as shown in the decrease of the band intensities in SDS-PAGE caused by HHP^30^,^31^ may change the combining capacity of IgE and IgG. The degraded protein fragments or the released peptides from squid Hc may destroy the binding sites and reduce the IgE and IgG values^32^. The immune reactivity of soybean seeds also decreased by HHP treatment at 300 MPa for 15 min^29^. The allergenicity of the bovine gamma globulin was decreased by HHP treatment (100–600 MPa at 5–7 °C for 5 min), because of the changes in the tertiary structure caused by HHP^9^. Nevertheless, the activity of phytoferritin (iron release activity) dramatically increased after the HHP treatment^26^. In this study, HHP caused a significant change in the protein structure of squid Hc by unfolding protein, decreasing FSC, and increasing Ho, thus decreasing the allergenicity. However, 600 MPa pressure did not lead further unfolding or change in FSC and Ho compared to 400 MPa. These modifications in protein conformations affected IgE-reactive conformational epitopes and hence changed allergenicity^6^,^7^. These results indicated that HHP treatments decreased the allergenic properties of squid Hc, and 400 MPa pressure was sufficient to control the allergenic properties.

In respect to established mathematical model, the best fit model was obtained when R^2^ was the highest, and χ^2^, RMSE, and MRD were the lowest. In general, when MRD was below 10%, indicating a good fit for practical purposes, and when RMSE was less than 10%, showing an excellent fit. The values of R^2^, χ^2^, MRD, and RMSE in this study were 0.98, 0.0051, 4.64%, and 6.30%, respectively, indicating that the model was good enough for evaluation^40^. The mathematical model established in this study was based on the information described by Ivanciuc *et al*.^41^, in which the relationship between hemocyanin structure and allergenicity index responses to HHP was modelled. This structure-allergenicity model could be used for explaining the complex changing process in the allergenic protein.

This study provided meaningful information of applying HHP to reduce allergenicity by changing protein structures in squid Hc. However, the exact mechanisms involved in the changes of the allergenicity and the structural properties induced by HHP should be studied further.

## Methods

### Materials

Squids (*T. pacificus*; 310 ± 25 × 10^−3^ kg per whole squid) were obtained from the Chinese Academy of Fishery Sciences (Shanghai, China) and stored at −80 °C until use. Individual human serum samples were collected from five patients (Xinhua Hospital, Shanghai, China) with squid allergy. The protocol was approved by the National Natural Science Foundation Commission of China (Permit Number: 31271955) and the Committee on the Ethics of Animal Experiments of School of Agriculture and Biology, Shanghai Jiao Tong University (SJTU). The rabbit anti-squid Hc polyclonal antibodies were obtained according to our previous methods^7^. All the procedures concerning animals were performed in accordance with the recommendations of the Guide for the Care and Use of Laboratory Animals of SJTU.

### Extraction, purification, and identification of Hc

The extraction, purification, and identification of squid Hc were carried out according to Coates *et al*.^3^ with some modifications, and all the procedures were carried out at 4 °C. In brief, the squid blood was extracted and mixed with an anti-coagulant buffer (5:1, v/v) containing 450 × 10^−3^ mol/L NaCl (pH 7.3), 10 × 10^−3^ mol/L KCl, and 10 × 10^−3^ mol/L EDTA. The mixture was centrifuged at 11,300 r/min for 50 min to precipitate the impurities. The supernatant was collected and subjected to dialysis against purified water for 36 h to remove small molecule proteins. Then, fractionation with a 67% (NH_4_)_2_SO_4_ solution was carried out overnight, followed by centrifugation at 11,300 r/min for 60 min. The collected protein precipitate was dissolved in Tris-HCl (1:1, w/v) containing 50 × 10^−3^ mol/L Tris (pH = 7.3) and 20 × 10^−3^ mol/L CaCl_2_. After the protein solution was dialysed against purified water for 12 h, the pure target protein was obtained. The protein concentration of the extracts was estimated using a BCA Protein Assay Kit (Beyotime, Shanghai, China) using bovine serum albumin as the standard. The obtained proteins were identified by SDS-PAGE and further confirmed by LC-Q-TOF-MS (Impact Q-TOF ultimate3000, Bruker Co., Germany).

### HHP treatments

The squid Hc solution was diluted with 20 × 10^−3^ mol/L Tris-HCl (pH 7.3) to 80 mg/mL. Then, 20 mL samples of the diluted squid Hc solution were individually packaged in polyamide/chlorinated polypropylene film bags (17 cm × 23 cm) for HHP treatments^37^. The HHP treatments were carried out using an HHP device (Kefa High Pressure Food Processing Inc., Baotou, China) at 200 MPa, 400 MPa, or 600 MPa for 20 min. A squid Hc sample held at ambient pressure (0.1 MPa) and temperature (25 °C) without HHP treatment was used as the control. The treatment conditions employed in this study were based on our previous studies^6^,^7^. Water was used as the pressure-transmitting medium, and the pressure vessel was maintained at ~25 °C. The pressure increase rate was 4.3 MPa/s, and the depressurization time was < 4 s. The pressure vessel (9 cm diameter and 32 cm height) had a 2 L volume. After the HHP treatments, all the samples were stored at the same −80 °C freezer and were diluted into certain concentrations for further analysis.

### Electrophoresis

The squid Hc samples (80 mg/mL) were analysed by SDS-PAGE performed following previous studies^7^ using 20% polyacrylamide slab gels. All the samples were boiled for 5 min before the electrophoresis, and the gels were stained with 0.1% Coomassie Brilliant Blue R-250. The standard protein used for MW determination was HiMark™ Pre-Stained Protein Standard (LC5699, Thermo Scientific, Massachusetts, USA).

### FTIR Spectra

The FTIR spectra of squid Hc were recorded using an IRPrestige-21 spectrometer (Nicolet 6700, Thermo Fisher, Waltham, MA, USA). The procedures applied for sample preparation and data processing were based on the method described by Haque *et al*.^21^. All protein samples were freeze-dried in a Freezone 2.5 L Triad system (Labconco Inc., USA), and 2 mg of protein powder was mixed with KBr, ground, and then pressed into a pellet. Absorbance intensity of the pellet was measured at 2 cm^−1^ resolution in a wavenumber range of 4000–400 cm^−1^. The measurements were repeated six times, and the values were averaged to reduce the baseline effects. Omnic V8.1 (ThermoFisher Scientific, USA) was used to smooth for spectra, and Peak Fit 4.12 (SeaSolve Software Inc., USA) was used to deconvolve the amide I region. The deconvolved spectrum was then iteratively curve-fitted with Gaussian band shapes. The resultant peaks were assigned to different secondary structures. Peak assignment of deconvolved amide I bands was conducted using the results of Haque *et al*.^21^ and Prosa *et al*.^22^ as guideline. Peaks at 1654 ± 4 cm^−1^, 1620 ± 20 cm^−1^, 1680 ± 20 cm^−1^ and 1645 ± 5 cm^−1^ corresponded to α-helix, β-sheet, β-turn and random coils, respectively, in the fitting procedure.

### FSC

The FSC was determined according to a modified method^36^ for tertiary structure analysis. Briefly, 1 mL of a squid Hc solution at a concentration of 1 mg/mL was mixed with 2 mL of 0.086 M Tris-Gly buffer (pH 8.0) containing 0.09 mol/L Gly, 0.004 mol/L EDTA, and 8 mol/L urea. A 0.02 mL of Ellman’s reagent (4 mg/mL DTNB in the Tris-Gly buffer) was then added to the mixture. After the incubation at 25 °C for 30 min, the absorbance at 412 nm was measured using a UV-1800 spectrophotometer (Shimadzu Co., Japan). The FSC was calculated as follows:


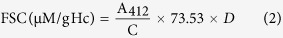


where FSC was expressed as μM/g Hc of squid; A_412_ was the absorbance of the sample; D was the dilution coefficient (3.02); C (mg/mL) was the protein concentration in the tested sample. Three replications were carried out.

### Ho

The Ho of squid Hc was determined using ANS as the fluorescence probe according to Hu *et al*.^25^ for tertiary structure analysis. In brief, serial dilutions in 10 × 10^−3^ mol/L PB (pH 7.0) were carried out with the Hc samples to a final concentration of 0.05–0.2 mg/mL, and 10 μL ANS (8. × 10^−3^ mol/L) prepared in the same buffer was added to 2 mL of sample. Fluorescence intensity was recorded at wavelengths of 390 nm (excitation) and 470 nm (emission) using an F-4500 FL spectrophotometer (Hitachi Co., Japan). The initial slope of the fluorescence intensity vs. protein concentration plot was used as the index of Ho. The measurements were performed in triplicate.

### SAXS

SAXS experiments were carried out using a Kratky-type camera (SAXSess, Anton Paar GmbH) following the modified method of Spinozzi *et al*.^14^ for quaternary structure analysis. Cr Ka1 radiation (2.2897E) was used as the incident beam equipped with an anode generator operated at 30 mA and 25 kV. The 2D data conversion to 1D and analysis of the SAXS curves were performed using the SAXS Quant 2.0 (Anton Paar, GmbH). The SAXS data were analysed using the SAXS Quant 2.0 and evaluated according to the following formulas^14^,^15^,^16^:


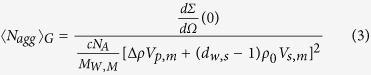



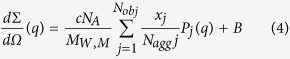



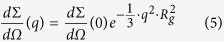



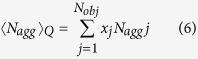


where 〈N_agg_〉_Q_ was the average aggregation number; 〈N_agg_〉_G_ was the Guinier aggregation number; 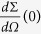 was the forward scattering cross-sections; R_g_ was the particle gyration radius. These four SAXS parameters were calculated for the analysis. All the basic data were available from the SAXS based on previous studies^13^,^14^,^15^. For example, c was the w/v protein concentration; N_A_ was the Avogadro number; M_w,m_ was the MW of the monomer; *Δρ* was the difference between the scattering length density of the protein (*ρ*_*p*_) and solvent (ρ_0_); d_w,s_ was the relative mass density of the hydration water, 1.10; and V_p,m_ and V_s,m_ were the estimated values of the core monomer volume and its first solvation shell, respectively.

### Allergenicity analysis

All experiments described in this study were performed in accordance with the recommendations of the Guide for the Care and Use of Laboratory Animals and Humans of Shanghai Jiao Tong University (SJTU). The protocols were approved by the Institutional Review Board of SJTU and National Natural Science Foundation Commission of China (Permit Number: 31271955). Blood collection was performed under anesthesia, and efforts were made to minimize suffering. Written informed consent was obtained from the subject. An indirect ELISA with the human sera of five allergic patients (P_1_–P_5_) was performed to analyse the allergenicity of the squid Hc samples following our previous method^7^. Moreover, indirect ELISAs with rabbit anti-squid Hc polyclonal antibodies (R_0_) for hydrolysates after the simulated gastric fluid (SGF) digestion (R_SGF_) and simulated intestinal fluid (SIF) digestion (R_SIF_) were performed. An automated ELISA plate reader (Thermo Co., USA) was used to monitor the absorbance at 450 nm. Three replicate measurements were carried out.

### Statistical analysis

Three replications were applied for each treatment. The results were reported as the mean ± standard deviation (SD). The statistical differences between different treatments were assessed using analysis of variance, followed by Tukey’s HSD post-hoc test (*P* < 0.05) using SAS 9.2 software (SAS Institute Inc., Cary, NC, USA). The mathematical model was developed by using MATLAB software (The Mathworks, Natick, MA, USA). The suitability of the model for the experimental and predicted data was determined using the correlation coefficient (R^2^), chi-square (χ^2^), MRD, and RMSE as described in the literature^40^:


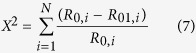



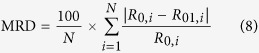



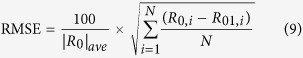


where *R*_*0*_ and *R*_01_ were the experimental and predicted data of the indirect ELISA performed using rabbit anti-squid Hc polyclonal antibodies, respectively, and N was the number of data points.

## Additional Information

**How to cite this article**: Zhang, Y. *et al*. Structure-based modelling of hemocyanin allergenicity in squid and its response to high hydrostatic pressure. *Sci. Rep.* **7**, 40021; doi: 10.1038/srep40021 (2017).

**Publisher's note:** Springer Nature remains neutral with regard to jurisdictional claims in published maps and institutional affiliations.

## Supplementary Material

Supplementary Information

## Figures and Tables

**Table 1:** Identification of squid hemocyanin by search engine MASCOT with LC-Q-TOF-MS protein fit list.

Rank	Scores	NCBI gi #	Species	Protein name	Protein MW (kDa)
1	2568.56	88657469	*Sepia officinalis*	Hemocyanin subunit 2	382.10
2	965.14	88657467	*Sepia officinalis*	Hemocyanin subunit 1	382.90
3	630.79	6685487	*Enteroctopus dofleini*	Hemocyanin G-type	331.70
4	449.49	346987844	*Helix lucorum*	Hemocyanin alpha N-subunit	345.90
5	408.22	56710669	*Nautilus pompilius*	Haemocyanin precursor	338.10
6	332.90	47169655	*Megathura crenulata*	Keyhole limpet hemocyanin1	358.70
7	306.89	47169657	*Megathura crenulata*	Keyhole limpet hemocyanin2	391.30
8	242.11	346987842	*Helix lucorum*	Hemocyanin alpha D-subunit	392.60
9	219.41	57335436	*Nucula nucleus*	Hemocyanin isoform 1	387.80
10	57.23	206716836	*Nierstraszella lineata*	Hemocyanin fg	62.50

**Table 2:** SAXS parameters of squid hemocyanin treated with different high hydrostatic pressures.

Treatment Group^+^	R_g_	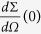	〈N_agg_〉_G_	〈N_agg_〉_Q_
Control	142.66 ± 2.52^a^	28.67 ± 1.53^a^	10.20 ± 0.78 ^c^	9.30 ± 1.14^c^
200	134.33 ± 1.15^b^	18.34 ± 1.43^b^	11.91 ± 1.26^bc^	12.67 ± 0.47^b^
400	121.33 ± 1.53^c^	14.67 ± 0.58^c^	14.20 ± 1.65^ab^	14.44 ± 1.01^a^
600	112.34 ± 2.52^d^	11.54 ± 1.55^d^	16.03 ± 1.01^a^	15.23 ± 0.98^a^

^+^200, 400, or 600: high hydrostatic pressure treatment at 200 MPa, 400 MPa, or 600 MPa for 20 min, respectively.

R_g_: particle gyration radius; 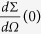: the forward scattering cross-sections; 〈N_agg_〉_G_: Guinier aggregation number; 〈N_agg_〉_Q_: average aggregation number. Means in the same column with different lowercase letters (a–g) are significantly different (*p* < 0.05).

**Table 3:** Results of the indirect ELISAs performed with the squid hemocyanin samples against IgE and IgG.

Pressure (MPa)	IgE binding*					IgG binding#		
	P_1_	P_2_	P_3_	P_4_	P_5_	R	R_SGF_	R_SIF_
0.1	1.35 ± 0.05^a^	0.81 ± 0.02^a^	0.77 ± 0.03^a^	0.53 ± 0.05^a^	0.44 ± 0.03^a^	1.37 ± 0.06^a^	1.20 ± 0.07^a^	0.50 ± 0.03^a^
200	1.07 ± 0.05^b^	0.50 ± 0.02^b^	0.47 ± 0.03^b^	0.29 ± 0.05^b^	0.27 ± 0.04^b^	1.17 ± 0.05^b^	0.91 ± 0.03^b^	0.33 ± 0.02^b^
400	0.93 ± 0.02^c^	0.31 ± 0.02^c^	0.30 ± 0.03^c^	0.22 ± 0.01^c^	0.21 ± 0.01^c^	1.06 ± 0.03^c^	0.83 ± 0.02^c^	0.22 ± 0.03^c^
600	0.93 ± 0.03^c^	0.32 ± 0.03^c^	0.31 ± 0.02^c^	0.21 ± 0.02^c^	0.21 ± 0.01^c^	1.06 ± 0.02^c^	0.83 ± 0.02^c^	0.27 ± 0.02^c^

Values were expressed as mean ± standard deviation (SD) (n = 3); means in the same column with different lowercase letters (a–c) are significantly different *(p* < 0.05).

^*^P_1_–P_5_: indirect ELISAs performed with the human sera of five allergic patients.

^#^R: indirect ELISA performed with rabbit anti-squid hemocyanin polyclonal antibodies. R_SGF_: indirect ELISA performed with rabbit anti-squid hemocyanin polyclonal antibodies for hydrolysates after the simulated gastric fluid (SGF) digestion. R-_SIF_: indirect ELISA performed with rabbit anti-squid hemocyanin polyclonal antibodies for hydrolysates after the simulated intestinal fluid (SIF) digestion.
