# Supplementary material for: Structure-based modelling of hemocyanin allergenicity in squid and its response to high hydrostatic pressure
Source: Sci Rep. 2017 Jan 23;7:40021. doi: 10.1038/srep40021 (PMC5256278; doi:10.1038/srep40021)
Supplement: Supplementary Information [file srep40021-s1.doc]

**Structure-based modelling of hemocyanin allergenicity in squid and its response to high hydrostatic pressure**

Yifeng Zhang^a^, Yun Deng^a*^, Yanyun Zhao^ab^

^a^ Key Laboratory of Urban Agriculture (South), Ministry of Agriculture, SJTU-Bor S. Luh Food Safety Center, Department of Food Science and Technology, Shanghai Jiao Tong University, 800 Dongchuan Road, Shanghai 200240, China

^b^ Department of Food Science & Technology, Oregon State University, 100 Wiegand Hall, Corvallis, OR 97331, USA

***Corresponding author:**

Mailing Address:

Dr. Yun Deng

Shanghai Jiao Tong University, 800 Dongchuan Road, Shanghai 200240, China

Tel: +86-21-34205755, Fax: +86-21-34205755

E-mail: foodsjtu@sjtu.edu.cn

**Supplementary information**

**Effect of different high hydrostatic pressures on peptide segments of squid hemocyanin**

**Results**

Effect of different high hydrostatic pressures on peptide segments of squid hemocyanin by liquid chromatography-tandem mass spectra (LC-MS) analysis are shown in Supplementary Table S1. In squid Hc samples, 27 types of peptide segments information (PS_1_-PS_27_) were selected from peptide mass fingerprint database for analysis. For PS_1_-PS_16_, there was no difference among all the samples. However, PS_17_ (SVDGAVLDPNSLPKPSLIYAPAK) and PS_18_ (AMQDDEGASGYQAISAYHGEPADCK) were changed by 600 MPa HHP treatment, result in SVDGAVLDPNSLPK and AMQDDEGASGYQAISAY respectively. For PS_19_-PS_27_, there were also differences between the control and HHP treated samples. PS_19_ and PS_20_ were disappeared in 600 MPa HHP treated samples. PS_21_ was changed by 400 and 600 MPa HHP treatments, resulted in undetected segments in those samples. However, PS_22_ -PS_27_ were disappeared in all HHP treatments. The results in Supplementary Table S1 revealed that peptide segments information were changed by HHP and protein cleavage was caused by a higher pressure.

**Methods**

The Liquid chromatography-tandem mass spectra (LC-MS) analysis was carried out for peptide segments analysis, using the Nano-Liquid Chromatography (NanoLC ,UltiMate3000 RSLCnano Liquid Chromatography, Bruker Daltonics Germany) and Quadrupole-Time-of-Flight Mass Spectrometer (Q-TOFMS, maXis impact UHR-TOF MS, Thermofishe, USA). The Hc samples were digested by pancreatic enzymes, and the small peptides (< 10 KD) were dislodged by elution. After the digestion and elution, the samples were injected into the LC-MS analysis. The LC-MS spectra were extracted and disposed by the SwissProt database (Release 2016 01). The peak list was directly generated from the raw data without peak smoothening or filtering. Mascot (Version 2.4, Matrix Science, USA) was set to search the National Center of Biotechnology Information (NCBI) database. The selective peptides information were used for analysis.

**Supplementary Table S1** Effect of different high hydrostatic pressures on peptide segments of squid hemocyanin by liquid chromatography-tandem mass spectra (LC-MS) analysis.

|  | Treatment Group | | | |
| --- | --- | --- | --- | --- |
| Peptide segments | Control | 200 | 400 | 600 |
| PS_1_ | DISSLTTEEEYELR | DISSLTTEEEYELR | DISSLTTEEEYELR | DISSLTTEEEYELR |
| PS_2_ | FDSLSFSGLSIPR | FDSLSFSGLSIPR | FDSLSFSGLSIPR | FDSLSFSGLSIPR |
| PS_3_ | FSGAIDSIGASTSR | FSGAIDSIGASTSR | FSGAIDSIGASTSR | FSGAIDSIGASTSR |
| PS_4_ | IPPSAQNLEVAIPLNR | IPPSAQNLEVAIPLNR | IPPSAQNLEVAIPLNR | IPPSAQNLEVAIPLNR |
| PS_5_ | IWAIWQALQK | IWAIWQALQK | IWAIWQALQK | IWATWQALQK |
| PS_6_ | KNVNSLTPSEIENLR | KNVNSLTPSEIENLR | KNVNSLTPSEIENLR | KNVNSLTPSEIENLR |
| PS_7_ | LFLYDISK | LFLYDISK | LFLYDISK | LFLYDISK |
| PS_8_ | LINEATFYNSR | LINEATFYNSR | LINEATFYNSR | LINEATFYNSR |
| PS_9_ | LSLVENELLAR | LSLVENELLAR | LSLVENELLAR | LSLVENELLAR |
| PS_10_ | LWAIWQDLQR | LWAIWQDLQR | LWAIWQDLQR | LWAIWQDLQR |
| PS_11_ | NVDSMTVSEMNALR | NVNSLTPSEIENLR | NVNSLTPSEIENLR | NVNSLTPSEIENLR |
| PS_12_ | SLPILVTEPK | SLPILVTEPK | SLPILVTEPK | SLPILVTEPK |
| PS_13_ | SPFSLASDINPDAMTR | SPFSLASDINPDAMTR | SPFSLASDINPDAMTR | SPFSLASDINPDAMTR |
| PS_14_ | TLQIEPNPFFK | TLQIEPNPFFK | TLQIEPNPFFK | TLQIEPNPFFK |
| PS_15_ | VFAGFWLSGIK | VFAGFWLSGIK | VFAGFWLSGIK | VFAGFWLSGIK |
| PS_16_ | SINGYQALAEFHGLPAK | SINGYQALAEFHGLPAK | SINGYQALAEFHGLPAK | SINGYQALAEFHGLPAK |
| PS_17_ | SVDGAVLDPNSLPKPSLIYAPAK | SVDGAVLDPNSLPKPSLIYAPAK | SVDGAVLDPNSLPKPSLIYAPAK | SVDGAVLDPNSLPK |
| PS_18_ | AMQDDEGASGYQAISAYHGEPADCK | AMQDDEGASGYQAISAYHGEPADCK | AMQDDEGASGYQAISAYHGEPADCK | AMQDDEGASGYQAISAY |
| PS_19_ | YVSLVEDELLAR | YVSLVEDELLAR | YVSLVEDELLAR | - |
| PS_20_ | SLASDINPDAMTR | SLASDINPDAMTR | SLASDINPDAMTR | - |
| PS_21_ | AVLGGETEMPWAFDR | AVLGGETEMPWAFDR | - | - |
| PS_22_ | DVQNLMSALKR | - | - | - |
| PS_23_ | EGSHFSVLGGSTEMPWAFDR | - | - | - |
| PS_24_ | NLLDFEDGEMESLR | - | - | - |
| PS_25_ | VAGGNEFFVGSIAVLGGS | - | - | - |
| PS_26_ | KPMSPFSLASDINPDAMTR | - | - | - |
| PS_27_ | GHNAIHSWVGGPS | - | - | - |

200, 400, or 600: high hydrostatic pressure treatment at 200 MPa, 400 MPa, or 600 MPa for 20 min, respectively.

PS_1_- PS_27_: different kinds of peptide segments.

-: not detected.
